# Treatment with an Anti-CX3CL1 Antibody Suppresses M1 Macrophage Infiltration in Interstitial Lung Disease in SKG Mice

**DOI:** 10.3390/ph14050474

**Published:** 2021-05-17

**Authors:** Satoshi Mizutani, Junko Nishio, Kanoh Kondo, Kaori Motomura, Zento Yamada, Shotaro Masuoka, Soichi Yamada, Sei Muraoka, Naoto Ishii, Yoshikazu Kuboi, Sho Sendo, Tetuo Mikami, Toshio Imai, Toshihiro Nanki

**Affiliations:** 1 Department of Internal Medicine, Division of Rheumatology, Toho University School of Medicine, Ota-ku, Tokyo 143-8541, Japan; satoshi.mizutani@med.toho-u.ac.jp (S.M.); junko.nishio@med.toho-u.ac.jp (J.N.); kano.kondo@med.toho-u.ac.jp (K.K.); kaori.motomura@med.toho-u.ac.jp (K.M.); zento.yamada@med.toho-u.ac.jp (Z.Y.); shoutarou.masuoka@med.toho-u.ac.jp (S.M.); soichi.yamada@med.toho-u.ac.jp (S.Y.); seimuraoka@med.toho-u.ac.jp (S.M.); 2 Department of Immunopathology and Immunoregulation, Toho University School of Medicine, Ota-ku, Tokyo 143-8540, Japan; 3 KAN Research Institute, Inc., Chuo-ku, Kobe-shi, Hyogo 650-0047, Japan; n-ishii@kan.eisai.co.jp (N.I.); y-kuboi@kan.eisai.co.jp (Y.K.); t-imai@kan.eisai.co.jp (T.I.); 4 Department of Internal Medicine, Division of Rheumatology and Clinical Immunology, Kobe University Graduate School of Medicine, Chuo-ku, Kobe-shi, Hyogo 650-0017, Japan; sho1000d@med.kobe-u.ac.jp; 5 Department of Pathology, Toho University School of Medicine, Ota-ku, Tokyo 143-8540, Japan; tetsuo.mikami@med.toho-u.ac.jp

**Keywords:** rheumatoid arthritis, interstitial lung diseases, CX3CL1/fractalkine, CX3CR1, M1 macrophage, M2 macrophage, SKG mice

## Abstract

CX3C Motif Chemokine Ligand 1 (CX3CL1; fractalkine) has been implicated in the pathogenesis of rheumatoid arthritis (RA) and its inhibition was found to attenuate arthritis in mice as well as in a clinical trial. Therefore, we investigated the effects of an anti-CX3CL1 monoclonal antibody (mAb) on immune-mediated interstitial lung disease (ILD) in SKG mice, which exhibit similar pathological and clinical features to human RA-ILD. CX3CL1 and CX3C chemokine receptor 1 (CX3CR1), the receptor for CX3CL1, were both expressed in the fibroblastic foci of lung tissue and the number of bronchoalveolar fluid (BALF) cells was elevated in ILD in SKG mice. No significant changes were observed in lung fibrosis or the number of BALF cells by the treatment with anti-CX3CL1 mAb. However, significantly greater reductions were observed in the number of M1 macrophages than in M2 macrophages in the BALF of treated mice. Furthermore, CX3CR1 expression levels were significantly higher in M1 macrophages than in M2 macrophages. These results suggest the stronger inhibitory effects of the anti-CX3CL1 mAb treatment against the alveolar infiltration of M1 macrophages than M2 macrophages in ILD in SKG mice. Thus, the CX3CL1-CX3CR1 axis may be involved in the infiltration of inflammatory M1 macrophages in RA-ILD.

## 1. Introduction

Rheumatoid arthritis (RA) is a systemic inflammatory disease that is characterized by synovitis, progressive bone erosion, and cartilage destruction [1]. Interstitial lung disease (ILD) develops in 10% of patients with RA, with alveolar septal fibrosis occurring through unknown mechanisms and resulting in more impaired pulmonary gas exchange and shorter life expectancy than in RA patients without ILD [2,3]. Limited information is currently available on the pathogenesis of RA-ILD and its exacerbating factors and an effective therapy has not yet been established [4]. 

ILD is characterized by abnormal tissue repair after lung tissue damage by chronic inflammation regardless of the cause. Macrophages, which are the most abundant immune cell in the lungs, play a key role in the development of ILD. Activated macrophages are polarized into classically activated M1 macrophages and alternatively activated M2 macrophages [5,6]. In the early inflammatory phase, M1 macrophages produce proinflammatory factors, such as tumor necrosis factor (TNF)-α, interleukin (IL)-1β, and inducible nitric oxide synthase [7,8,9,10,11]. In contrast, M2 macrophages contribute to tissue fibrosis by secreting profibrotic cytokines, including IL-4, IL-10, and transforming growth factor-β [12,13,14,15]. Lung macrophages are also classified into alveolar and interstitial macrophages depending on their locations, both of which are involved in the pathogenesis of ILD [16,17]. Although embryonic alveolar macrophages dominant in the steady state, the infiltration of alveolar macrophages derived from circulating monocytes also occurs in murine bleomycin-induced ILD (BLM-ILD) [17]. Monocyte-derived alveolar macrophages, but not embryo-derived resident alveolar macrophages, express profibrotic genes in BLM-ILD [16], suggesting that these migrated monocyte-derived alveolar macrophages are responsible for ILD. 

The sole member of the CX3C-type chemokine family, chemokine (C-X3-C motif) ligand 1 (CX3CL1; fractalkine) is a membrane-bound chemokine that is expressed on a number of cells, including endothelial cells, fibroblast-like synoviocytes, osteoblasts, neurons, adipocytes, and intestinal epithelial cells. [18]. The extracellular domain of CX3CL1 is constitutively cleaved and functions as a soluble chemokine [19,20]. In the steady state, circulating monocytes expressing CX3C chemokine receptor 1 (CX3CR1), the receptor for CX3CL1, adhere to endothelial cells through the CX3CL1-CX3CR1 interaction in order to monitor vascular abnormalities [19]. Membrane-bound CX3CL1 is more strongly expressed on endothelial cells under inflammatory conditions, leading to the firm adherence and activation of CX3CR1^+^ monocytes that initiate local inflammation. The cleavage of membrane CX3CL1 is also promoted by TNF-α, IL-1, and interferon-γ. The recruitment of CX3CR1-expressing inflammatory cells, including monocytes, NK cells, cytotoxic T cells, type 1 helper T cells, and γδ T cells, has been shown to exacerbate local inflammation [21]. 

Proliferative fibroblast-like synoviocytes express CX3CL1 and inflammatory cells, such as macrophages and T cells, express CX3CR1 in RA joints [22,23,24,25]. We previously demonstrated that the blockade of CX3CL1 efficiently suppressed collagen-induced arthritis in mice [25]. A clinical trial on a humanized anti-CX3CL1 monoclonal antibody (mAb) for RA reported clinical efficacy for active RA [26,27]. These findings indicate that the CX3CL1-CX3CR1 axis contributes to the progression of RA through the CX3CL1-dependent migration of activated macrophages into the synovium. 

Limited information is currently available on the role of the CX3CL1-CX3CR1 axis in RA-ILD. CX3CL1 is expressed on alveolar and bronchial epithelial cells and vascular endothelial cells in the normal lungs of humans and mice [28,29]. In idiopathic pulmonary fibrosis (IPF) and ILD in polymyositis or dermatomyositis (PM/DM), CX3CL1 is expressed on fibroblasts, inflammatory cells, and alveolar macrophages in addition to the epithelia and vessels [28,29]. Serum CX3CL1 levels were found to correlate with the alveolar-arterial oxygen pressure difference in patients with ILD with PM/DM [30]. On the other hand, CX3CR1^+^ mononuclear cells infiltrated ILD in patients with systemic sclerosis [31] and PM/DM [30] as well as murine BLM-ILD [32,33]. In BLM-ILD, the depletion of CX3CR1-expressing cells suppressed lung fibrosis and this was accompanied by a decrease in infiltrated macrophages [32]. These findings implicate the CX3CL1-CX3CR1 axis in lung fibrosis.

SKG mice develop RA-like chronic polyarthritis and ILD following an injection of zymosan A [34,35]. Since the point mutation of zeta-chain-associated protein kinase 70 (ZAP-70) in SKG mice promotes autoreactive T-cell development in the thymus, these articular and lung manifestations are considered to develop through autoimmune mechanisms. While arthritis develops in the early stages (4–5 weeks) after the administration of zymosan A, lung inflammation manifests later and fibrosis becomes evident after approximately 12 weeks [34,36]. Therefore, ILD in SKG mice (SKG-ILD) is considered to more closely reflect RA-ILD than other ILD models. 

In the present study, we investigated the involvement of CX3CL1 and its inhibition in ILD in SKG mice. A treatment with anti-CX3CL1 mAb suppressed the infiltration of M1 macrophages, but not M2 macrophages, into the alveolar space, but did not attenuate lung fibrosis, suggesting the potential of CX3CL1 to regulate macrophage infiltration in SKG-ILD.

## 2. Results

### 2.1. Histopathological Findings of SKG-ILD

**Figure 1:**
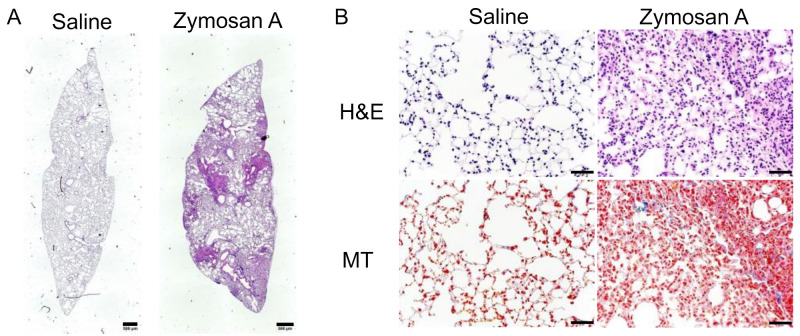
Interstitial lung disease (ILD) induced in SKG/jcl (SKG) mice. ILD was induced in male SKG mice by an intraperitoneal injection of 7.5 mg zymosan A or saline as the control at 8–9 weeks of age. Lung tissue was isolated 12 weeks after the administration of saline or zymosan A and stained with hematoxylin and eosin (H&E) and Masson’s trichrome (MT). (**A**) A representative image of whole area of longitudinal H&E section of the lung from SKG mice administered saline or zymosan A at × 40 magnification. Scale bars indicate 500 μm. (**B**) Images of H&E (upper panels) or MT (lower panels) staining at × 200 magnification. Scale bars indicate 50 μm.

SKG mice spontaneously develop ILD at approximately 6 months of age under conventional conditions [34], but not under specific-pathogen-free (SPF) conditions [36]. Therefore, we induced ILD in SKG mice under SPF conditions at 8 weeks of age using the intraperitoneal administration of zymosan A. We confirmed that lung tissues collected 12 weeks after the administration of zymosan A exhibited multiple fibroblastic foci that were fibrotic areas with mononuclear cell infiltration and a consequently altered alveolar structure, while those from mice administered saline showed no signs of inflammation (Figure 1A,B). Masson’s trichrome (MT) stain showed collagen deposition colocalized with infiltrating cells in fibroblastic foci (Figure 1B), which confirmed that SKG mice administered zymosan A developed robust ILD in our SPF mouse facility. 

### 2.2. Expression of CX3CL1 and CX3CR1 in Lungs with ILD in SKG Mice

**Figure 2:**
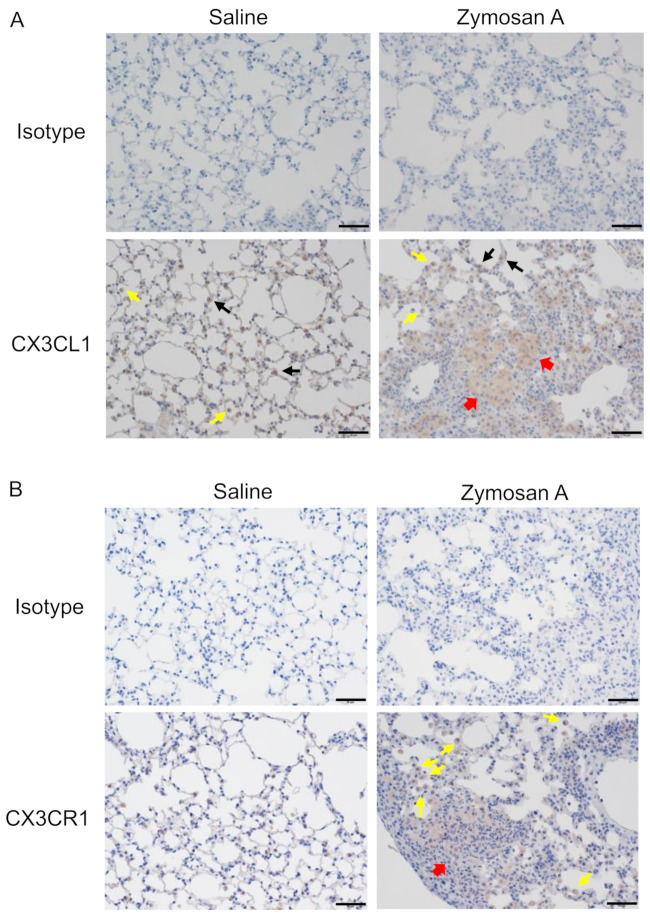
Immunohistochemical (IHC) analysis of CX3CL1 and CX3CR1 in lung tissue from SKG-ILD mice. Lung tissue was obtained as described in Figure 1. Representative images of the IHC analysis of CX3CL1 or CX3CR1 are shown. (**A**) Images of IHC for CX3CL1. Arrows indicate CX3CL1-positive alveolar epithelial cells (black arrows), alveolar macrophages (yellow arrows), or CX3CL1-stained areas in fibroblastic foci (red arrows). (**B**) Images of IHC for CXC3R1. Arrows indicate CX3CR1-positive alveolar macrophages (yellow arrows) or CX3CR1^+^ cell-infiltrating areas in fibroblastic foci (red arrows). Original magnification of ×200. Scale bars indicate 50 μm.

To examine the involvement of CX3CL1 in the pathogenesis of ILD in SKG mice, an immunohistochemical analysis of the expression of CX3CL1 and CX3CR1 in lungs with ILD was performed (Figure 2). While CX3CL1 was expressed on alveolar epithelial cells and macrophages in lungs from both saline-(control) and zymosan A-administered mice, CX3CL1 appeared to accumulate in the fibroblastic foci of lungs in zymosan A-administered mice (Figure 2A, the 3,3′-diaminobenzidine (DAB) staining intensities in control and zymosan A-administered mice, 0.015 vs. 11.136). CX3CR1 was only expressed on alveolar macrophages, the number of which was small, in control mice, whereas CX3CR1-expressing cells massively infiltrated fibroblastic foci in zymosan A-administered mice (Figure 2B, the DAB staining intensities in control and zymosan A-administered mice, 0.094 vs. 5.552). Although alveolar macrophages appeared to be present in the fibrotic foci of lungs with ILD, the destruction of the alveolar structure made them less distinct. These results suggested the involvement of the CX3CL1-CX3CR1 axis in the infiltration of alveolar macrophages and interstitial macrophages in SKG-ILD. 

### 2.3. Minimal Effects of the Blockade of CX3CL1 in the Lung Pathology of SKG-ILD

**Figure 3:**
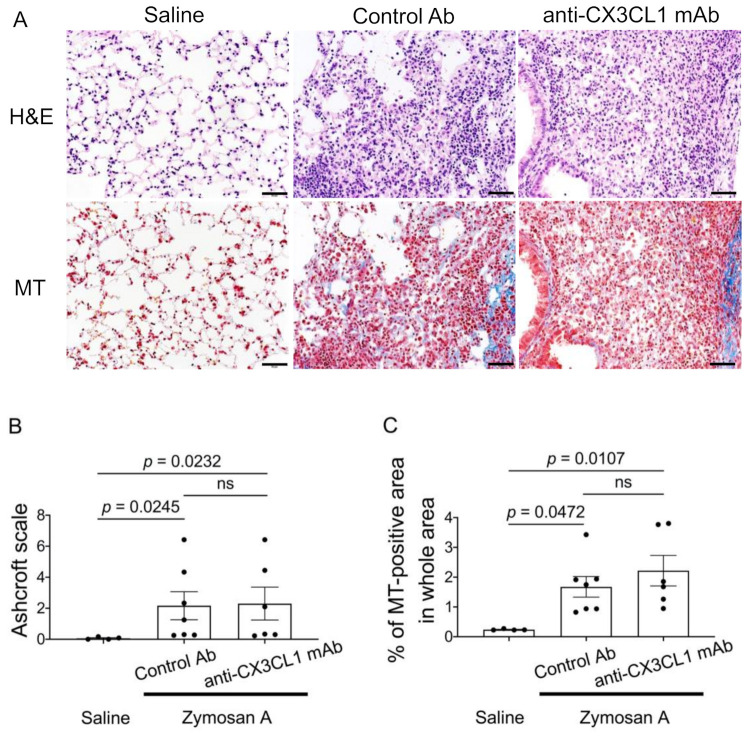
No significant changes in lung fibrosis by the anti-CX3CL1 mAb treatment in SKG-ILD. SKG mice were treated with an intraperitoneal injection of control Ab (hamster immunoglobulin) (*n* = 7) or anti-CX3CL1 mAb (*n* = 6) twice a week for 12 weeks immediately after the administration of zymosan A until euthanization. (**A**) Representative images of lung tissues stained with H&E (upper panels) or MT (lower panels). Original magnification × 200. Scale bars indicate 50 μm. (**B**) The Ashcroft scale was used to assess H&E-stained lung tissues. (**C**) The percentage of MT-positive (blue color-stained) areas in the whole area. The black points indicate each sample value. Data are expressed as means ± standard error of the mean (SEM). ns, not significant. The Kruskal–Wallis test was used with Dunn’s test as a post hoc test.

Based on the results showing that CX3CR1^+^ cell numbers increased in the lung tissue of SKG-ILD, we hypothesized that the migration of CX3CR1-positive cells contributes to lung inflammation and fibrosis. To address this hypothesis, we treated SKG-ILD mice with neutralizing anti-CX3CL1 mAb or a control antibody (Ab) and assessed the lung histology of ILD. Anti-CX3CL1 mAb was administered twice a week from the day of the administration of zymosan A until mice were euthanized. Lung tissue from control Ab- and anti-CX3CL1 mAb-treated mice both showed fibroblastic foci with massive cell infiltration and an altered alveolar structure (Figure 3A). The accumulation of collagen bundles was also similarly observed in lungs from control Ab-treated mice and anti-CX3XCL1 mAb-treated mice with MT staining (Figure 3A). No significant differences were noted in Ashcroft scores [37] or collagen-deposited areas between control Ab-treated mice and anti-CX3CL1 mAb-treated mice (Figure 3B,C). These results indicated that the inhibition of CX3CL1 had minimal effects on fibrotic changes in the lungs of SKG-ILD. 

### 2.4. Flow Cytometric Analysis of Bronchoalveolar Fluid (BALF) Cells in SKG-ILD

**Figure 4:**
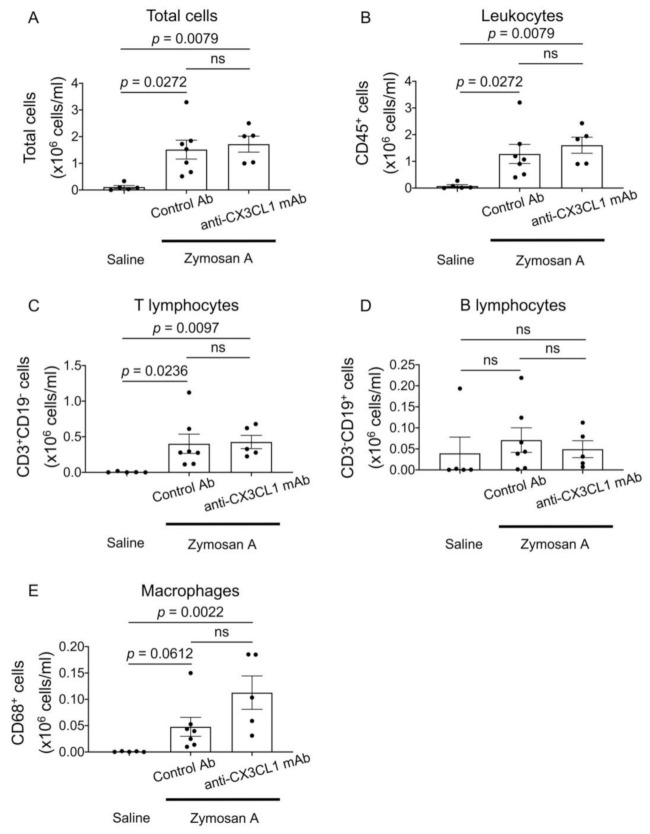
No significant changes in numbers of individual immune cell populations in BALF from SKG-ILD mice treated with anti-CX3CL1 mAb. BALF cells were isolated from saline-administered SKG mice (*n* = 5) or zymosan A-administered SKG mice treated with control Ab (*n* = 7) or anti-CX3CL1 mAb (*n* = 5). The numbers of all cells (**A**), CD45^+^ cells (**B**), T lymphocytes (**C**), B lymphocytes (**D**), and macrophages (**E**) are shown. Since 4 mL of saline was used to obtain BALF, total cell numbers of individual populations are estimated by multiplying the concentration (cells/mL) by 4 mL. Data are expressed as means ± SEM. The Kruskal–Wallis test was used with Dunn’s test as a post hoc test.

Although the inhibition of CX3CL1 only negligibly affected lung fibrosis, marked changes were observed in CX3CR1^+^ cells that had abundantly infiltrated the alveolar space. We performed a flow cytometric analysis of BALF cells in SKG-ILD to investigate changes in alveolar cell populations following the treatment with anti-CX3CL1 mAb. The numbers of all cells, leukocytes, and T lymphocytes in BALF were significantly higher in SKG-ILD mice than in control saline-injected SKG mice. Furthermore, the number of CD68^+^ macrophages was markedly higher in SKG-ILD mice than in control SKG mice. No changes were observed in BALF B lymphocytes following the induction of ILD. However, the administration of anti-CX3CL1 mAb did not significantly alter the numbers of these cell populations (Figure 4).

### 2.5. Effects of the Blockade of CX3CL1 on Alveolar Macrophages in SKG-ILD

**Figure 5:**
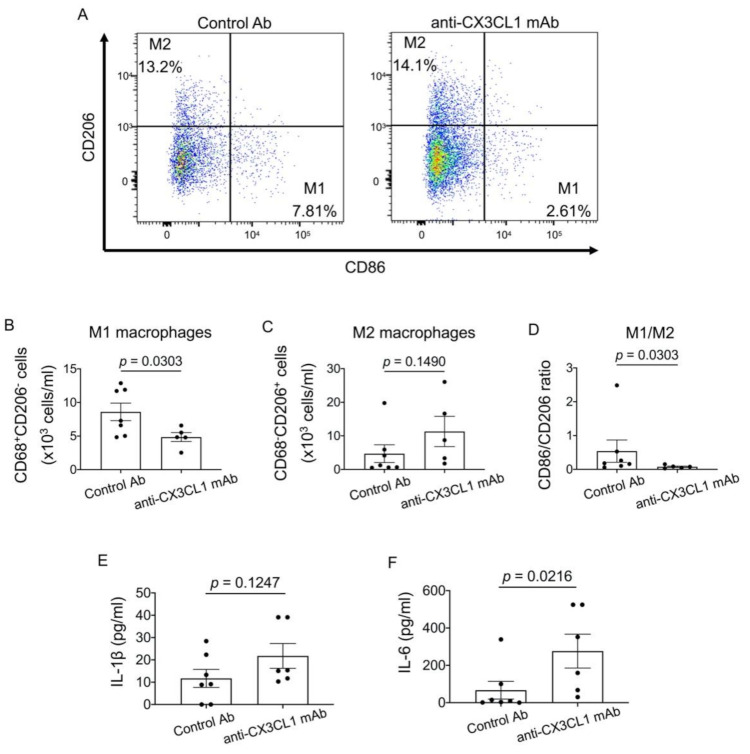
Alterations in M1 and M2 macrophage numbers in BALF following the treatment with anti-CX3CL1 mAb. BALF cells obtained in Figure 4 were analyzed for M1 and M2 macrophages. (**A**) Representative flow cytometry scatter plots for the expression of CD86 and CD206 in CD68^+^ macrophages. (**B**–**D**) The numbers of M1 macrophages (CD86^+^CD206^−^ cells; (**B**) and M2 macrophages (CD206^+^CD86^−^ cells; (**C**) and the M1/M2 ratio (**D**) are shown. (**E**,**F**) Levels of interleukin (IL)-1β (**E**) and IL-6 (**F**) in BALF. Data are expressed as means ± SEM. The Mann–Whitney U test was performed.

Since macrophages play a critical role in ILD, we examined M1 (CD86^+^CD206^−^) and M2 (CD206^+^CD86^−^) macrophages in BALF. The number of M2 macrophages was similar between control Ab-treated mice and anti-CX3CL1 mAb-treated mice (Figure 5A,C), which is consistent with the lack of an effect of the anti-CX3CL1 treatment on fibrosis. In contrast, the number of M1 macrophages significantly decreased following the anti-CX3CL1 mAb treatment (Figure 5B), and consequently the M1/M2 ratio significantly decreased (Figure 5D), suggesting skewed polarization toward M2 macrophages. However, the level of IL-1β in BALF was not altered and IL-6 in BALF rather increased following the anti-CX3CL1 mAb treatment (Figure 5E,F). Thus, these results indicate that anti-CX3CL1 mAb inhibited M1 macrophage infiltration and skewed polarization toward M2 macrophages, consistently with little anti-fibrotic effects of the blockade of CX3CL1.

### 2.6. High Expression Levels of CX3CR1 in Alveolar M1 Macrophages

**Figure 6:**
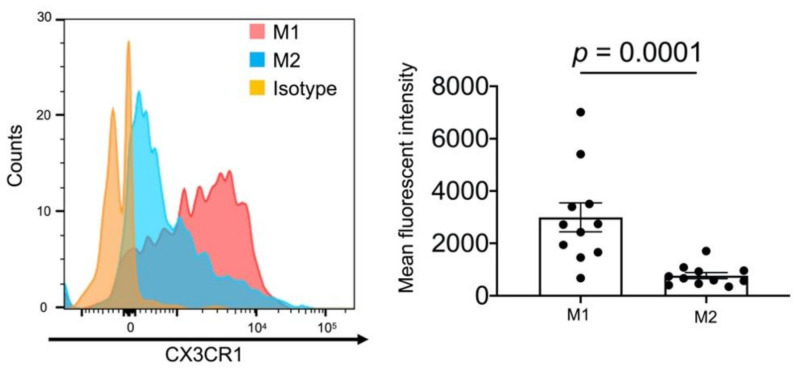
Expression of CX3CR1 on M1 or M2 macrophages. BALF cells obtained from SKG-ILD mice were analyzed for the expression of CX3CR1 on M1 and M2 macrophages. (**A**) Representative histogram of CX3CR1 expression on CD45^+^CD68^+^CD86^+^CD206^−^-gated cells (M1) or CD45^+^CD68^+^CD86^−^CD206^+^-gated cells (M2). (**B**) Pooled data on the mean fluorescent intensity of CX3CR1 expression on M1 or M2 macrophages (*n* = 11). Data are expressed as means ± SEM. The Mann–Whitney U test was conducted.

We examined CX3CR1 expression levels on BALF M1 and M2 macrophages in SKG-ILD mice. Although M1 and M2 macrophages both expressed CX3CR1, its expression levels were significantly higher in M1 macrophages than in M2 macrophages (mean fluorescent intensity; M1, 2994 ± 551.6, M2, 767.8 ± 117.9, Figure 6). This result suggested that the higher expression level of CX3CR1 more strongly inhibited the alveolar infiltration of M1 macrophages by anti-CX3CR1 mAb than that of M2 macrophages. 

## 3. Discussion

In the present study, we administered a treatment with anti-CX3CL1 neutralizing mAb to SKG mice with immune-mediated ILD. The results obtained demonstrated that CX3CL1^+^ and CX3CR1^+^ cells localized to fibroblastic foci in SKG-ILD and that the anti-CX3CL1 mAb treatment reduced the number of M1 macrophages in BALF. However, the treatment did not significantly alter the number of BALF M2 macrophages or fibrosis in SKG-ILD. We also found that CX3CR1 expression levels in BALF were higher in M1 macrophages than in M2 macrophages in SKG-ILD, suggesting that anti-CX3CL1 mAb more strongly inhibited the migration of M1 macrophages than M2 macrophages. 

Although the contribution of the CX3CL1-CX3CR1 axis to the pathogenesis of ILD has already been demonstrated using a murine BLM-ILD model, its involvement in immune-mediated ILD remains unclear. The present study is the first to examine the role of the CX3CL1-CX3CR1 axis in immune-mediated ILD using SKG mice. The expression of CX3CL1 and CX3CR1 in lung fibroblastic foci was observed in SKG-ILD mice, similar to the murine BLM-ILD model [29,32,33]. CX3CR1^+^ macrophages that localize to fibrotic loci have been shown to promote fibroblast migration or proliferation through the production of platelet-derived growth factor-AA in BLM-ILD [32]. CX3CL1 and CX3CR1 are abundantly expressed in ILD in patients with RA (unpublished data). These findings indicate that the CX3CL1-CX3CR1 axis is involved in the pathogenesis of RA-ILD, similar to the murine model of ILD.

Anti-CX3CL1 mAb therapy reduced the number of BALF M1 macrophages, but not M2 macrophages, in SKG-ILD, and only negligibly attenuated lung fibrosis in SKG-ILD. Based on the result showing that BALF M1 macrophages expressed a significantly higher level of CX3CR1 than BALF M2 macrophages in SKG-ILD, we speculated that anti-CX3CL1 mAb efficiently suppressed the migration of M1 macrophages, but not M2 macrophages. 

The minimal effects of anti-CX3CL1 mAb on SKG-ILD is consistent with our recent findings showing the negligible effects of anti-CX3CL1 mAb therapy on the number of infiltrated BALF M2 macrophages in a BLM-ILD model [29]. In contrast, previous studies reported that genetically CX3CR1-depleted mice were resistant to BLM-ILD regardless of whether they were congenic or inducible-deficient mice [32,33]. Therefore, the use of CX3CL1-blocking antibodies may have different outcomes from the complete genetic absence of the CX3CL1-CX3CR1 signal. 

ILD is characterized by abnormal tissue repair during chronic inflammation. Although M2 macrophages are considered to play a pivotal role in the process of fibrosis, M1 macrophages are also necessary for inflammation eliciting abnormal tissue repair. In the present study, anti-CX3CL1 mAb successfully inhibited the migration of M1 macrophages, but failed to suppress the migration and/or polarization of M2 macrophages; therefore, it did not exert therapeutic effects against lung fibrosis with CX3CL1 blockade alone. Moreover, the levels of IL-1β and IL-6 in BALF did not decrease following the anti-CX3CL1 mAb treatment. These imply that anti-CX3CL1 mAb treatment could not dampen IL-1β and IL-6 production even though it reduced M1 macrophages in BALF. This is probably partially because these cytokines were also produced by activated fibroblasts and/or the other macrophages in uncontrolled lung fibrosis.

The present study had several limitations that need to be addressed. Although we observed a decrease in BALF M1 macrophages in SKG-ILD following the treatment with anti-CX3CL1 mAb, the number of BALF cells reflects, but may not directly contribute to, inflammation and/or fibrosis in the lung. Therefore, the effects of anti-CX3CL1 mAb on lung-infiltrating cells remain unclear. Another limitation is that anti-CX3CL1 mAb was administered at the same time as the zymosan A injection. In SKG mice, lung fibrosis becomes evident several weeks after an injection of zymosan A. A different treatment outcome may have been observed if anti-CX3CL1 mAb had been administered once lung fibrosis was established.

In summary, our study demonstrated that the CX3CL1-CX3CR1 axis contributed to the pathogenesis of ILD through the migration of CX3CR1^+^ cells into inflammatory lung tissue expressing CX3CL1 in SKG mice, a model of RA-ILD. Although anti-CX3CL1 mAb therapy did not attenuate lung fibrosis in SKG-ILD, the infiltration of BALF M1 macrophages strongly expressing CX3CR1 into the lungs was efficiently suppressed. Although anti-CX3CL1 mAb alone did not exert therapeutic effects against lung fibrosis, its combination therapy with anti-fibrotic drugs, such as nintedanib or pirfenidone, may be expected to have a therapeutic effect on ILD.

## 4. Materials and Methods

### 4.1. SKG Mice

Male SKG/jcl mice were purchased from CLEA Japan Inc (Tokyo, Japan). SKG/jcl mice aged 8–9 weeks (*n* = 11) were intraperitoneally administered 7.5 mg of zymosan A (Alfa Aesar, Lancashire, UK) dissolved in 0.5 mL of physiological saline. Control mice (*n* = 5) were administered 0.5 mL of saline. Mice were euthanized 12 weeks after the administration of zymosan A to assess pulmonary fibrosis. Regarding the treatment with anti-CX3CL1 mAb, 500 μg of anti-CX3CL1 mAb (5H8-4) [25,38] was intraperitoneally injected twice a week for 12 weeks from the day of the zymosan A administration. Hamster Ig [39] was used as the control Ab for the control group of mice. All experimental procedures were performed in the SPF animal facility of Toho University. Animal experiments were performed according to the animal experiment guidelines approved by Toho University Animal Care and User Committee (approved number: #18-51-398, approved date: 24 May 2018).

### 4.2. Histopathological Investigation

Mice were euthanized with an overdose of injectable anesthetics 12 weeks after the administration of zymosan A. Left lung tissue was fixed with 10% neutral formalin solution and embedded in paraffin. Three-micrometer-thick sections were used for hematoxylin and eosin (H&E) staining, the MT stain for collagen deposition, and an immunohistochemical analysis. In the immunohistochemical analysis, sections were stained with rabbit immunoglobulin as the isotype control (Dako X0903, Santa Clara, CA, USA), rabbit anti-CX3CL1 polyclonal Ab (pAb, Boster PA1401, Pleasanton, CA, USA), or anti-CX3CR1 (Abcam ab8021, Cambridge, UK,) after blocking endogenous peroxidase and consequent blocking with 2.5% goat serum. An incubation with the primary Ab was conducted at room temperature for 3 h for anti-CX3CL1 pAb or for 30 min for anti-CX3CR1 pAb. The ImmPRESS polymer kit (Vector MP-7451-15, Burlingame, CA, USA) was used to detect Ab staining, and counterstaining with hematoxylin was performed. All histological images were captured using a BX-63 microscope (Olympus, Tokyo, Japan).

To assess lung fibrosis, ten fields under the × 40 view were randomly selected from each H&E section, scored using the Ashcroft scale [37], and the average score of each section was calculated [37]. Regarding collagen quantification, MT-stained areas and the total cross-section area were quantified by ImageJ software (National Institute of Health) and the percentage of MT areas in the total area was calculated. To assess the intensity of CX3CL1 or CX3CR1 staining in IHC images, the mean value of DAB staining per pixel in the lung interstitial area after thresholding was calculated by ImageJ.

### 4.3. Flow Cytometric Analysis of BALF

BALF was obtained as previously described [29]. Briefly, 1 mL of saline with 100 µM EDTA was intratracheally injected and aspirated by a 24-G catheter with a 1 mL syringe. This procedure was repeated 4 times and recovered BALF was pooled. 

After Fc blocking with 20 µg/mL of rat anti-mouse CD16/CD32 mAb (2.4G2, BD, Franklin Lakes, NJ, USA), BALF cells were stained with PE-conjugated rat anti-mouse CD68 mAb (FA-11, BioLegend, San Diego, CA, USA), BV421-conjugated rat anti mouse CD86 mAb (GL-1, BioLegend), APC-conjugated rat anti mouse CD206 mAb (C068C2, BioLegend), BV510-conjugated rat anti-mouse CD3 mAb (17A2, BioLegend), FITC-conjugated rat anti-mouse CD19 mAb (1D3/CD19, BioLegend), APC/Cy7-conjugated rat anti-mouse CD45 mAb (30-F11, BioLegend), and PE/Cy7-conjugated rat anti-mouse CX3CR1 mAb (SA011F11, BioLegend). A flow cytometric analysis was performed using BD LSRFortessaTM (BD Biosciences, San Jose, CA, USA) and data were analyzed using FlowJo software ver. 10.7.1 (BD Biosciences).

### 4.4. Enzyme-Linked Immunosorbent Assay (ELISA) for BALF

Supernatant of BALF were collected and stored at −80 °C until subjected to the assay. ELISA was performed to measure the levels of IL-1β and IL-6 using mouse IL-1β ELISA kit (Proteintech KE10003, Rosemont, IL, USA) and mouse IL-6 quantikine ELISA kit (R&D systems 6000B, Minneapolis, MN, USA), respectively by following manufactural protocols.

### 4.5. Statistical Analysis

Statistical analyses were performed using Graph Pad Prism ver. 8.3.1 (Graph Pad Software, San Diego, CA, USA). The Kruskal–Wallis test was used to compare three groups. Dunn’s test was employed as a post hoc test. The Mann–Whitney U test was conducted to compare two groups. A p-value less than 0.05 was considered to be significant. Results were shown as the mean ± SEM.

## 5. Conclusions

Our study suggests that the CX3CL1-CX3CR1 axis contributes to the pathogenesis of RA-ILD through the migration of CX3CR1+ cells. Anti-CX3CL1 mAb therapy efficiently suppressed the infiltration of BALF M1 macrophages in the mouse model of RA-ILD, and therefore, this therapy combined with anti-fibrotic drugs may have a more robust therapeutic effect on lung fibrosis.

## Data Availability

The data presented in this study are available on request from the corresponding author.
